# Prevalence of probable post-traumatic stress disorder and experiences of trauma in emerging adults living with HIV in Zimbabwe

**DOI:** 10.1192/bjo.2024.720

**Published:** 2024-12-13

**Authors:** Renato Silveira, Sainath Eleti, Emily Saruchera, Rukudzo Mwamuka, Susannah Whitwell, Melanie A. Abas, Helen E. Jack

**Affiliations:** Institute of Psychiatry, Psychology and Neuroscience, King's College London, London, UK; Faculty of Life Sciences and Medicine, Kings College London, London, UK; Faculty of Medicine, University of Zimbabwe, Harare, Zimbabwe; Maudsley Hospital, South London and Maudsley NHS Foundation Trust, London, London, UK; Division of General Internal Medicine, Department of Medicine, University of Washington School of Medicine, Seattle, USA.

**Keywords:** Post-traumatic stress disorder, people living with HIV, low- and middle- income countries, Zimbabwe, emerging adults

## Abstract

**Background:**

Little is known about the prevalence of post-traumatic stress disorder (PTSD) in emerging adults living with HIV in low-income countries.

**Aims:**

Determine prevalence of trauma exposure, prevalence of probable PTSD and conditional prevalence of probable PTSD for different traumatic events; and better understand the experiences of individuals with HIV and PTSD.

**Method:**

This mixed method study used secondary data from a cross-sectional survey of people (*N* = 222) aged 18 to 29 living with HIV in Zimbabwe and primary qualitative data collection. The PTSD Checklist for DSM-5 (PCL-5) and the Life Events Checklist for DSM-5 (LEC-5) were used to measure PTSD and exposure to traumatic events, both translated to Shona. In-depth interviews (*n* = 8) with participants who met the criteria for probable PTSD were analysed using thematic analysis.

**Results:**

In all, 68.3% [95% CI (61.4–74.1)] of participants reported exposure to at least one traumatic event. The observed prevalence of probable PTSD was 8.6% [95% CI (5.2–13.0)], most observed following exposure to fire or explosion 29.0% [95% CI (13.0–45.0)] and sexual assault 27.8% [95% CI (7.2–48.7)]. Probable PTSD was also more prevalent following multiple exposure to trauma; four and six events, *N* = 4 (21%) [95% CI (5.1–8.8)] each, two and three events *N* = 3 (15.7%) [95% CI (5.9–9.2)] each, and five events *N* = 1 (5.4%) [95% CI (7.5–9.6)]. Qualitative results indicated that HIV stigma exacerbated psychological distress from trauma.

**Conclusions:**

Despite trauma exposure being common, prevalence of probable PTSD was not high, but was higher in those with multiple exposures. Participants described coping strategies, including social support and religious thinking.

The prevalence of HIV in Zimbabwe is 12.7%, one of the highest in the world.^1^ HIV and poor mental health are mutually reinforcing, with mental health contributing to worse HIV outcomes.^2^ HIV has been associated with increased risk to self (suicidality and self-harm),^3^ substance use disorders^4^ and depression.^5^ In Zimbabwe, it has been shown that there is an increased risk of common mental health difficulties (CMD) such as anxiety and depression among people living with HIV (PLWH), compared with people who are HIV negative (67.9% versus 51.4%).^6^ Emerging adults, individuals aged 18–29, with HIV are particularly vulnerable to mental health conditions.^7^ This has been associated with the transition period from adolescence to adulthood which involves many potential interpersonal, social and economic challenges, including the greater risk of relative poverty.^8^ Despite this, this age group has been given little attention in the development of psychological interventions to address mental health conditions, and most existing research has focused on anxiety and depression. There has been little attention paid to post-traumatic stress disorder (PTSD).^7^

Populations in low- and middle-income countries (LMICs), such as Zimbabwe, are likely to be at greater risk of PTSD, as they are more frequently exposed to traumatic events associated with poverty than those in high income countries (HICs).^9^ Despite a higher exposure to traumatic events, protective factors may limit PTSD onset in LMIC populations. A study examining lay health workers in Zimbabwe found that only 6% reached the threshold for probable PTSD.^10^ Additionally, the availability of services for the screening and treatment of PTSD among PLWH in LMICs is limited. A recent study surveying a random sample of HIV clinics in several LMICs discovered that only 19% of sites screened for the disorder, 51% managed it and 14% did both.^4^ PTSD varies with the type of traumatic event^11,12^ and the number of traumatic events one is exposed to, as evidence suggests multiple exposure cumulatively increases risk of PTSD.^13^ The development of services to better serve adolescents and young adults living with HIV in LMICs must be guided by data on the magnitude of traumatic experiences and PTSD, and by their lived experiences of trauma and coping.

This mixed methods study aims to characterise experiences of trauma and probable PTSD among emerging adults living with HIV in Harare, Zimbabwe. The specific objectives of this study are to:

- Determine the prevalence of experiences of trauma, probable PTSD and conditional prevalence of probable PTSD associated with exposure to different traumatic events.
- Explore the perspectives of emerging adults living with HIV with probable PTSD on experiences of trauma, HIV and their coping strategies using in-depth interviews.

Improved understanding of the prevalence and nature of trauma and PTSD in this vulnerable population could inform efforts to support their mental health and engagement with HIV care.

## Method

### Study setting

The study was conducted at a public HIV clinic at a hospital in Harare, Zimbabwe. The clinic's services include HIV testing and treatment, with 5000 registered patients of all ages.

### Design and approach

The current study used a mixed methods design involving a combination of secondary data analysis from a large cross-sectional study and primary data collection of qualitative interviews with select participants from the larger study. The parent study explored barriers to adherence of antiretroviral therapy (ART) among young adults,^14^ and it was expanded to include primary qualitative data collection for the current study. The qualitative interviews were added to better understand participants’ experiences with HIV and PTSD, which could not be captured in the survey data. The quantitative methods within this study are reported according to the Strengthening the Reporting of Observational Studies in Epidemiology (STROBE) guidelines.^15^

### Cross-sectional study

#### Recruitment

Recruitment was conducted in the waiting room of the HIV clinic. To be included, participants had to be between 18 and 29 years of age and registered at the clinic, and they had to have started ART at least one month beforehand. Participants were excluded if clinical staff (doctors or nurses) thought they had severe physical or mental illness. Random sampling was used to select participants: emerging adults attending the clinic from Monday to Friday were assigned a number, which was placed into a container from where a research assistant (RA) randomly picked numbers. The aim was to sample 12 participants a day. If fewer than 12 emerging adults were attending appointments on that day, all of them were invited. A total of 268 individuals were approached. While 222 met inclusion criteria and agreed to take part; 11 did not meet inclusion criteria and 35 declined, which most reported was because of a lack of time.

#### Data collection

Trained RAs fluent in Shona and English verbally administered the questionnaires and all measures in one-to-one sessions in private rooms at the HIV clinic. Verbal administration of research questionnaires is standard procedure in Zimbabwe due to variable literacy, and several prior mental health studies have used this approach.^6,16^ Data were collected on paper and then entered into Excel by the RA.

#### Measurements

Demographic data collected included gender, highest level of education obtained and employment status. Additional self-report HIV-related information was HIV status, disclosure of HIV status to others, confirmation of ongoing ART treatment and mode of transmission.

Probable PTSD was measured using the Shona version of the PTSD Checklist for DSM-5 (PCL-5). We use the term ‘probable’ as the PCL-5 is a screening rather than a diagnostic tool, so it can only tell us that someone likely has PTSD, but it cannot give a certain diagnosis of PTSD. The PCL-5 is a self-report questionnaire that contains 20 questions to assess four symptom clusters: intrusion, avoidance, hyperarousal and negative alterations in cognition and mood. Participants completed a 5-point Likert scale in which 0 = Not at all and 4 = Extremely.^17^ The minimum possible score is 0, and the maximum possible score is 80. The Shona version of PCL-5 had been previously validated with good internal consistency (Cronbach's alpha = 0.92) and suggested that a threshold of 33 or higher had sensitivity of 74.5% for probable PTSD and specificity of 70.6% for no PTSD.^18^ For the current study, those scoring above 32 were considered to have screened positive and thus to have probable PTSD, consistent with the prior validation study.

Traumatic events were measured with the Life Events Checklist for DSM-5 (LEC-5), a self-report questionnaire including 16 potentially traumatic events (e.g. fire or explosion, sexual assault, life-threatening injury or illness) and an open category, ‘other’, to capture any other events the participants considered traumatic. Participants were asked to indicate if and how they experienced the event, varying on six different levels of exposure: happened to me, witnessed it, learned about it, part of my job, not sure and does not apply. For this study, only direct exposure (i.e. first-hand experiences) was considered exposure to trauma, while indirect (learning about it) was not, as the probability of PTSD following indirect exposure is lower.^19^ LEC-5 is considered a reliable tool to capture participants’ actual level of exposure to trauma and is used along with PLC-5 and Clinician-Administered PTSD Scale for DSM-V (CAPS-5) to establish a PTSD diagnosis.^20^ The current study used a validated Shona version of LEC-5.^18^ Zimbabwean researchers further adapted the questionnaire for the current study by removing events that do not commonly occur in a Zimbabwean context (e.g. exposure to toxic substance, natural disaster, war). This resulted in a shortened version with ten traumatic events and an open question, ‘other’.

#### Data analysis

The data were analysed using STATA version 17 for Mac. Initial procedures included eliminating duplicate entries, converting string variables into numerical format and consolidating variables to facilitate a more straightforward interpretation of results. We established a criterion based on the percentage of missing data for each record. Specifically, if a participant's record was missing more than 5% of its data, we removed the record entirely. Conversely, if the missing data for a participant's record were less than 5%, we retained the record. This level of missing data is deemed acceptable within the context of statistical analysis, allowing for robust and reliable analytical outcomes.^21^ We identified ten records missing one value and two missing two values on exposure to trauma according to LEC-5 variable. Imputation would lead to overreporting of participants not exposed to a given event. Therefore, for transparency we reported results only for those who responded and indicate the total number of responses to each variable.

Data from LEC-5 were combined to report proportions of direct exposure to different traumas. Overall prevalence of probable PTSD was obtained based on PCL-5 scores. A dichotomous categorical variable was created according to the PTSD threshold previously established for the Zimbabwean population: 0 = No PTSD for participants who scored lower than 33, and 1 = PTSD for those who scored 33 or higher.^16^

### Qualitative interviews

#### Sample

All participants who were surveyed and reached the threshold for probable PTSD (*n* = 19) on preliminary analysis of the PCL-5 were invited for a qualitative interview; and eight agreed to participate. Those who did not accept reported lack of time as the reason. The final sample was composed of six women and two men with a mean age of 24.6 years.

#### Data collection

Participants underwent two phases of interviews: free listing (Supplementary Appendix A available at https://doi.org/10.1192/bjo.2024.720) and semi-structured interviews. The free listing method involves asking participants to list all the items they can think of that relate to a specific topic. The free listing interviews elicited participants’ items on HIV, trauma and others in the community with similar experiences. The results of the free listing were used to inform the interview guide in the current study.^22^ This allowed for further exploration of participants’ own experiences and perceptions about other community members who may have shared similar challenges. Interviews lasted 1 to 2 h and took place in private rooms at the HIV clinic. They were conducted by RAs fluent in English and Shona, trained in ethnographic interviewing and without a prior relationship with any participant. Participants could opt to answer the questions in their preferred language. Interviews were digitally recorded, translated from Shona where applicable and transcribed for analysis.

#### Data analysis

The data analysis of in-depth interviews was guided by principles of thematic analysis.^23^ Code list development was carried out by a cross-cultural team of global mental health researchers and trainees from King's College London (H.E.J., M.A.A., R.S. and S.E.), some with extensive experience of working in Zimbabwe (M.A.A. and H.E.J.). Coding was performed inductively based on the understanding that participants were the specialists of their own experiences, allowing themes to emerge from data.^23^ Three interviews were individually coded by each researcher, then discussed in the team meeting where an initial code list was agreed. This initial code list was applied on the remaining interviews (S.E., E.S. and R.S.), and it was verified if it comprehensively covered participants’ experiences or if new codes were needed. A few adaptations to the initial code list were suggested, discussed and agreed in the team meeting, generating the final code list. Zimbabwean-based researchers who also collected the original data (E.S. and R.M.) reviewed and provided input on all stages of coding. All transcripts were coded individually by two researchers (R.S. and S.E.) based on the final code list. Individual codes were then compared, and any differences were reconciled through discussion. Examples of inductive codes included self-isolation, confidentiality/privacy, HIV stigma, HIV treatment adherence and social exclusion. The constant comparison method was used to refine codes and ensure consistency.^24^ Finally, like codes were grouped into themes to capture patterns in the data.

### Ethics

The initial project which provided data for the current secondary analysis received approval from the University of Cape Town Human Research Ethics Committee (652/2018), the Joint Parirenyatwa Hospital and College of Health Sciences Research Ethics Committee (JREC 297/18) and the Medical Research Council of Zimbabwe (B/1623). Participation was voluntary, detailed information about quantitative and qualitative phases of the study was provided, and written informed consent was obtained prior to the study. Data were completely anonymised prior to this secondary analysis. All participants excluded for being unwell and those who were included in the current study following positive screening for PTSD were referred to the Parirenyatwa Centre of Excellence for further assessment/treatment.

## Results

### Cross-sectional study

#### Sample description

The final sample included 222 participants. Missing data were below 5% across records; therefore, none were removed from analysis. The mean age was 22.3 years (s.d. = 3.2), and the majority (59.9%) were women (*n* = 133). Most had primary education or less (74%), and many were unemployed (39.6%). The majority 64.4% (*n* = 143) reported vertical infection (born with HIV), followed by 24.3% (*n* = 54) reporting non-vertical infection, and the remaining 11.3% (*n* = 25) reporting not knowing or declining to answer about the mode of infection. (See Table 1 for the full sample description.)

**Table 1:** Participants’ demographic characteristics

Demographic	Overall (*N* = 222)	
	*N*	%
Gender		
Male	89	40.1
Female	133	59.9
HIV mode of infection		
Vertical (born with HIV)	143	64.4
Non-vertical (intercourse, others)	54	24.3
Declined to answer	25	11.3
Orphanhood status		
Both parents alive	55	24.8
Lost father	52	23.5
Lost mother	44	19.8
Lost both	71	32.0
Marital status		
Married and/or cohabiting	46	20.7
Divorced and/or separated and/or widowed	24	10.8
Single and/or never married	152	68.5
Employment status		
Employed (part- and full-time)	41	18.5
Self-employed	29	13.1
Student	64	28.8
Unemployed	88	39.6
Educational level		
Primary education or less	166	74.8
Secondary education or more	56	25.3

#### Exposure to trauma measured by LEC-5

A total of 68.3% (*n* = 151) of participants reported experiencing or witnessing a traumatic event or encountering it as part of their job. The most common traumatic event participants reported experiencing was ‘life-threatening illness or injury’ (14.1%) followed by ‘physical assault’ (13.6%) and ‘transportation accident’ (10.9%). Additionally, 21.6% of participants used the open category ‘other’ to report an event they considered traumatic that was not covered by the LEC-5 (e.g. ‘I was accused of robbery’, ‘my divorce’). Nine participants stated that when they used the ‘other’ category, they were referring to HIV diagnosis as a traumatic event. A very small proportion (6.5%) reported a traumatic event as part of their job.

#### Prevalence of PTSD

Nineteen participants scored 33 or more on PCL-5, representing an overall prevalence of probable PTSD of 8.6% [95% CI (5.2–13.0)]. Probable PTSD prevalence among women was 10.5% [95% CI (8.1–19.9)] and 5.6% [95% CI (1.0–11.0)] among men. For participants with an education level of primary school or less, 10.2% [95% CI (5.4–14.6)] met the criteria for probable PTSD, whereas 3.5% [95% CI (−1.1–9.13)] did who had secondary school education or more.

#### Conditional prevalence of probable PTSD by traumatic event

At least one case of probable PTSD was observed after exposure to each type of traumatic event. PTSD was most common following ‘fire or explosion’ (29%), ‘sexual assault’ (27.8%), the open category ‘other’ traumatic events (25.4%) and sudden or accidental death (24.2%) (see Table 2 for the full list).

**Table 2:** Prevalence of probable post-traumatic stress disorder (PTSD) after exposure to different types of traumatic events in emerging adults living with HIV.

Traumatic event	Exposed^a^	PTSD (%)	95% CI
Transportation accident	78/221	18.0	(9.5–26.5)
Physical assault (being attacked, hit, slapped, kicked, beaten up)	61/222	18.3	(8.3–27.6)
Others	55/216	25.4	(13.5–36.4)
Serious accident (at work or home, or during recreational activity)	49/222	16.3	(5.7–26.3)
Life-threatening illness or injury	45/222	15.6	(5.3–26.7)
Sudden accidental death of someone close to you	33/222	24.2	(9.4–38.6)
Fire or explosion	31/222	29.0	(13.0–45.0)
Assault with a weapon (being shot, stabbed, threatened with a knife)	24/219	12.5	(−1.0 to 25.0)
Sexual assault (rape, attempted rape, any type of sexual act through force or threat)	18/222	27.8	(7.2–48.7)
Sudden violent death (e.g. homicide, suicide)	14/220	7.1	(−6.4 to 20.4)
Serious injury, harm or death you caused to someone else	6/222	16.7	(−13.0 to 47.1)

a. Denominators indicate the number of participants who answered this question on the Life Events Checklist for DSM-5.

#### Multiple exposure and probable PTSD

Of 222 participants, 199 were exposed to multiple traumatic events, whereas 11 were exposed to a single event and 12 were exposed to none. Eighty reported exposure to six events 36.0% [95% CI (8.2–22.2)], followed by 30 participants (13.4%) [95% CI (5.6–15.1)] with exposure to four events, 27 participants (12.10%) [95% CI (3.4–14.1)] were exposed to five events, the same number (27) was observed among those who experienced three traumatic events, 20 participants (9.00%) [95% CI (5.4–12.1)] to two events and 15 were exposed to more than six events.

**Fig. 1:**
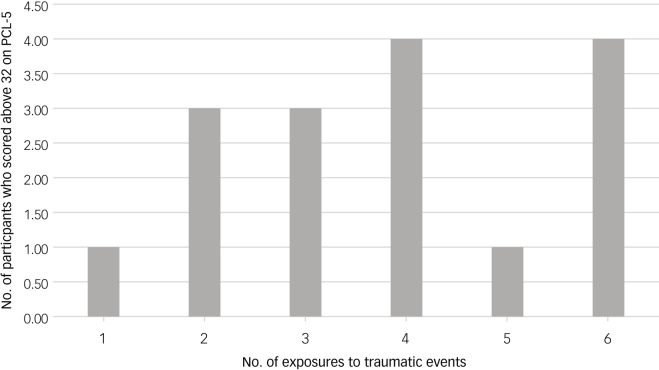
Prevalence of probable post-traumatic stress disorder by number of exposures to traumatic events.

Probable PTSD was mainly observed among those who experienced four and six traumatic events *N* = 4 (21%) [95% CI (5.1–8.8)] each, followed by two and three events *N* = 3 (15.7%) [95% CI (5.9–9.2)] each, and five events *N* = 1 (5.4%) [95% CI (7.5–9.6)]. One participant exposed to only one traumatic event met the criteria for probable PTSD (5.3%) [95% CI (7.5–9.6)]. Additionally, two participants who reported exposure to over six traumatic events met the criteria for probable PTSD, and one participant who reported no exposure to trauma met the criteria. Thus, a total of 19 participants met the criteria for PTSD (see Fig. 1).

### Qualitative results

Participants described both their own experiences and the experiences of others whom they perceived to have experienced trauma. Five themes emerged from data analysis: trauma experiences related to HIV status; physical manifestations of trauma; psychological expression of trauma; social isolation and lack of support; and coping and strategies for improving trauma care. Given the limited number of participants sampled the research team concluded that theoretical saturation was not reached.

#### Theme 1 – trauma experiences related to HIV status

Several participants explained that being diagnosed with and having HIV led to psychological distress, and interpersonal difficulties with their family members and society in general. Others spoke about the fear of potential HIV infection following an traumatic event such as rape, exacerbating the distress from that event:

‘*When I was almost raped by my stepfather, I was stressed a lot because you will think that what if he managed to rape me and yet he is HIV positive, and I was HIV negative by then.’* (Participant 25)

Several participants reported having difficulties adhering to their HIV treatment regimen, not because of any specific issues with the medication or adverse effects. Rather, they attributed this to distress from previous traumatic events and associated difficulties which led to a lack of motivation to live:

‘ *… if it was someone else who went through what [I] went through, that person could stop taking his or her medication.’* (Participant 45)

Furthermore, some participants spoke about ‘stigmatisation, discrimination and isolation’, which they attributed to their HIV positive status. Some thought they were isolated and discriminated against because they or people with whom they interacted did not understand HIV. For instance, one participant said:

‘*I can say, when I was tested at first, I had self-stigma especially at my home. I wouldn't want to share even a cup with my siblings.’* (Participant 45)

Overall, many participants faced psychological challenges related to acquiring or living with HIV, and had experienced potentially traumatic events related to their HIV status.

#### Theme 2 – physical manifestations of trauma

Many participants described physical responses in response to remembering prior traumatic events. These included headaches, restlessness, weight loss, sleeping difficulties and gastrointestinal issues:

‘*When I watch films in relation to the incident that happened to me [trauma], I become so restless that I cannot sit still.’* (Participant 136)

‘*Headaches, sometimes you just shiver.’* (Participant 22)

Additionally, many participants reported physical symptoms being triggered when experiencing discrimination, often related to past traumatic events or their HIV status:

‘*Personally, I lost weight because people were consistently talking about me, but with time, through going to the police and the clinic, I regained my weight.’* (Participant 9)

#### Theme 3 – psychological expression of trauma

Many participants stated that the social stigma and negative thinking that followed traumatic experiences caused them to cry, or feel ‘sad’, ‘lonely’, ‘angry’ or ‘worried’. Several participants described, and observed others, thinking ‘about a lot of things’ and ‘thinking deeply’ to the point that they were unable to focus on daily tasks. One participant described almost being run over by a vehicle while deep in thought:

‘*They will be thinking that they will not be able to get help. Some would have realised that no one is able to assist them to solve their problem so they will be thinking too much.’* (Participant 300)

Other participants described being easily startled, which caused them to act verbally and physically aggressively, even towards friends and family. Several participants also reported feeling an overall sense of helplessness and hopelessness, which made it challenging for them to cope with having HIV. A few described that these feelings led to thoughts of suicide:

‘*Someone can go to the extent of hurting themselves. Yes, I almost killed myself with rat poison.’* (Participant 64)

Similarly, some participants described not adhering to their HIV treatment either because they were preoccupied with their distressing trauma or as a way of ‘ending’ their suffering.

#### Theme 4 – social isolation and lack of support

All participants reported a preference for not sharing any information about their HIV status and traumatic experiences due to fear of social judgement, and many stated that this unwillingness to share resulted in self-isolation. Many mentioned that friends and family blamed them for their HIV status and traumatic experiences. Some participants noted that others made the participants start to blame themselves. A few participants described that they and others in their community were excluded from religious activities, school or workplaces:

*‘When people at work discovers that you are an HIV patient, they start to discriminate [against] you. Some will not even eat the food that you eat, some will stop being friends with you and some can call you names because they do not know how you got infected.’* (Participant 9)

For some, this contributed to financial hardship and worsened some participants’ already challenging relationship with family members:

*‘Then on the financial issue some family members may see no reason of taking care of you … Especially let's say a person have been raped.’* (Participant 300)

Overall, many participants were secretive about their past traumatic experiences and HIV status, believing that disclosure could lead to psychosocial difficulties.

#### Theme 5 – coping and strategies for improving trauma care

Many participants reported their own ways of dealing with the impacts of HIV and traumatic experiences. They noted that social support, faith and their own psychological strategies, such as repeating to themselves that all was well, were most helpful when they had negative thoughts.

‘*So, you would better leave it in God's hands, because even if they blame you, it won't change anything, as you know that you did nothing wrong.’* (Participant 9)

Many participants also spoke about external sources of support. They displayed broadly positive attitudes towards healthcare workers and counselling, particularly talk therapy. However, many also reported difficulties opening up to counsellors, as the counsellors were often from an older generation. Counsellors who were not relatable and the cost of counselling were the main barriers to engagement with mental health services. All participants shared possible strategies that might help alleviate their psychological distress such as (a) increasing the availability of counselling sessions for those with HIV, (b) reducing the impact of social isolation with the implementation of support groups and (c) provision of programmes in which participants could spend time outside of their community and thus avoid the numerous reminders of their trauma. Additionally, some participants emphasised the importance of improving education about HIV at a population level:

*‘Those people who abuse and isolate others should be impacted by the knowledge that being HIV+ doesn't mean the end of the world, and [they] still have a life to live. But if they don't have that knowledge the problem will keep on, on rise.’* (Participant 25)

## Discussion

This study reports the prevalence of probable PTSD for emerging adults (18–29 years old) living with HIV in an LMIC and stratifies prevalence of PTSD by type of traumatic event (conditional prevalence). We also used qualitative interviews to explore emerging adults’ lived experience of HIV and PTSD. While 68.3% of the sample reported exposure to trauma, only 8.6% met the criteria for probable PTSD, lower than in prior studies of PLWH in the region^16^ and globally.^25^ Participants who experienced more than one trauma had a higher prevalence of probable PTSD. The qualitative data revealed that complex and intersecting experiences of HIV stigma and discrimination affected participants’ exposure to trauma and ability to cope with trauma.

The prevalence of PTSD in this study is significantly lower than that found in another study of PLWH in Zimbabwe (probable PTSD 55.3%).^16^ The mean age of participants included in the prior study was 34, markedly older than in this study. The PTSD prevalence we found, however, is more similar to another recent study in Zimbabwe, which included lay health workers (LHWs) who provided support for a population who were HIV positive, trauma-exposed and/or with CMD diagnosis. The LHWs had a mean age of 62 (s.d. 8.3), and the study reported that only 6% sampled met criteria for probable PTSD and 11% for CMD.^10^ In another study of an HIV-positive cohort of younger individuals (12–24 years old) in Tanzania, the prevalence of probable PTSD was 10%,^26^ quite close to that found in this study. The disparity on PTSD prevalence might be explained by different levels of exposure and severity of traumatic events, as well as individual circumstances such as support network and individual characteristics. Moreover, PTSD is challenging to diagnose and screen for, particularly cross-culturally; some of the differences in prevalence across these studies may also be because of challenges in PTSD measurement, particularly using brief screening tools.

There are several studies exploring the resilience of emerging adults to potential sources of trauma.^27,28^ In the LMIC context, several important coping strategies have been identified, and they largely fall under three main themes: interpersonal (social support), intrapersonal (agency, appraisal of trauma) and spiritual (religion).^29^ The participants in our qualitative interviews discussed many of these coping strategies; while the participants in our qualitative sample had probable PTSD, others in their community who are HIV positive may use similar coping mechanisms, which may have played a role in preventing the development of PTSD among those who had experienced trauma. The qualitative results illustrate that many participants sought comfort in their faith, in the hope of finding someone who could provide support without judgement, and in external support such as talking therapy.

There are several factors that may limit our ability to generalise the prevalence of PTSD in our sample to the overall population of emerging adults in Zimbabwe. The most vulnerable individuals might not have taken part in this study. Our recruitment was conducted in a clinical setting (tertiary hospital) in the capital city, which means that we only sampled people who were accessing healthcare and living in an urban area. Several participants (35/268) recruited, who met inclusion criteria, declined to take part in this study, which may be because of a lack of time, possibly a consequence of poverty and social vulnerability. Additionally, the possibility of stigmatisation within the participants’ communities might have reduced willingness to participate or answer honestly.^30^ HIV stigma in Zimbabwe is known to reduce help-seeking behaviour.^31^ In fact, those who took part expressed their preference for secrecy about their HIV status and reported it as a barrier to engagement with services.

Importantly, in the current study, only one participant who was exposed to a single traumatic event met the criteria for probable PTSD. Most of those who met the criteria for probable PTSD had been exposed to two or more events. This finding is aligned with previous research that identified a cumulative impact of trauma on increased severity of PTSD symptoms in a sample of adolescents in South Africa.^13^ In HICs, a higher risk of developing PTSD was also observed among those who experience multiple exposures to trauma.^32^ Qualitative interviews in the current study showed how traumas may have compounded each other. For example, the risk of acquiring HIV after exposure to sexual violence can exacerbate the distress, stigma, blame and powerlessness associated with the sexual trauma itself. The stigma attached to HIV, whether the individual had confirmed infection or was at high risk of infection, led participants to report self-isolating and not reaching out for support, which might have reduced their ability to cope. Additionally, our findings are aligned with the theoretical understanding of HIV diagnosis as a potentially psychologically distressing experience, characterised by fear of the future and stigma, but not necessarily itself a trauma that leads to PTSD.^33^ Rather, we see that emerging adults living with HIV are at high risk of experiencing other types of trauma, only some of which are directly related to their HIV positive status, and that some who experience trauma go on to develop PTSD.

This study has several limitations. The measurement of PTSD was done using a screening tool rather than through clinical diagnosis with a mental health professional. Thus, we can only report probable PTSD rather than a confirmed PTSD diagnosis. A research assistant verbally administered the screening tool, which could have led to underreporting of PTSD symptoms due to stigma, shame or social desirability bias. Finally, qualitative interviews were conducted only among those who had probable PTSD, which limits our ability to draw conclusions about the coping strategies and protective factors of those who experienced trauma but did not have probable PTSD. Furthermore, many people with probable PTSD declined to participate, primarily because of the lack of time. Given the small number of people who met the inclusion criteria and agreed to participate, theoretical saturation was not reached. Given the paucity of data on PTSD in emerging adults in Zimbabwe and other LMICs, this exploratory qualitative study may inform hypotheses that could be tested in future quantitative studies with larger sample sizes or further explored through in-depth qualitative work.

### Future research

Symptoms of other psychopathologies were observed in qualitative assessments of the current study, including anhedonia, rumination and negative views about the self, others and the future. A higher prevalence of CMD (11%) over probable PTSD (6%) has been previously reported in a Zimbabwean sample.^10^ Therefore, the prevalence of depression, anxiety and substance use and its relationship with trauma in this population should be further explored, as exposure to trauma and other challenging experiences, such as HIV diagnosis, may lead to other psychopathologies such as depression, self-harm and suicidal ideation.^33,34^

Previous research in Zimbabwe has expanded the understanding of local expressions of depression symptoms such as *kufungisisa*; an expression used to describe ‘thinking too much’ which is closely aligned with the Western concept of rumination.^35^ Although the current study has uncovered some local expressions of trauma, more work is needed to fully understand how this population expresses and conceptualises PTSD symptoms. Importantly, nine participants reported HIV diagnosis as a traumatic event itself, and this should be further explored. Additionally, further research should better characterise traumatic events, such as the nature and causal factors of fires and explosions which were prevalent in this sample, to direct trauma prevention interventions.

Given the high prevalence of trauma exposure, research should focus on strategies to reduce exposure trauma in the first instance and methods of promoting protective factors among vulnerable populations, thus shifting the focus from treatment to prevention.

### Implications for practice

Many HIV patients in Zimbabwe are exposed to multiple traumatic events. Based on our qualitative interviews, the intersection of HIV and trauma was central to the lives of emerging adults living with HIV who have probable PTSD. To support these individuals, there is a need for trauma treatment that is integrated with HIV care to address this complex biopsychosocial intersection of HIV and trauma. Many participants expressed the need to have a safe space where they could talk with someone, and they specifically wanted that person to be relatable. Accordingly, interventions might be best delivered by peers or community health workers, who may be more relatable to patients and can be trained in basic counselling techniques.^36^ This is supported by recent evidence showing peer interventions to be effective in reducing barriers to accessing care, predominantly through decreasing stigma.^37^ Efforts to develop or scale-up peer-delivered interventions, however, need to consider how the peers will be supervised, trained and supported to work with people who have experienced trauma and PTSD. Overall, there is a great need to consider context and culturally sensitive interventions^38^ to address the unique needs of emerging adults with HIV who have experienced trauma. The high prevalence of people who have experienced trauma also speaks to the need to invest in efforts to prevent the development of PTSD. This could potentially be accomplished through reducing the incidence of trauma with strategies such as HIV stigma reduction in society and healthcare settings^39^ and parenting programmes to reduce violence at earlier stages of life.^40^ Other potential strategies require additional research, but might include topics such as a change in attitudes toward gender norms and domestic violence, poverty reduction, and programmes to reduce road accidents through improved traffic safety.

Emerging adults are at a critical period of their life, where they are vulnerable to the development of psychiatric disorders and poor HIV treatment outcomes.^7^ The observed psychological distress of this population may derive not only from PTSD symptoms and HIV treatment challenges, but may stem from deeply rooted societal processes, including HIV stigma, violence and poverty. However, despite considerable exposure to trauma, relatively few had probable PTSD, suggesting important coping strategies and/or protective factors in this population. More work is needed to understand how to reduce exposure to trauma, the negative mental health effects of trauma and PTSD in this population.

## Supporting information

Silveira et al. supplementary material Silveira et al. supplementary material

## Data Availability

The data that support the findings of this study are available from the corresponding author, M.A.A., upon reasonable request.
