# Supplementary material for: Prevalence of probable post-traumatic stress disorder and experiences of trauma in emerging adults living with HIV in Zimbabwe
Source: BJPsych Open. 2024 Dec 13;11(1):e7. doi: 10.1192/bjo.2024.720 (PMC11733456; doi:10.1192/bjo.2024.720)
Supplement: Silveira et al. supplementary material [file S2056472424007208sup001.docx]

**Appendix A: Free listing interview guide**

*1. Have you experienced nightmares or intrusive memories, been easily startled or on edge or*

*avoided places or people since your traumatic experience?*

*2. What are the problems of people who have had traumatic experiences (such as assault or seeing*

*a loved one die) as well as living with HIV in your community?*

Probe until the interviewee is not able to list any further problems

If the problem described relate to FEELINGS, THOUGHTS, BEHAVIOURS or

RELATIONSHIPS:

*a) Who is knowledgeable about these problems in the community?*

*b) Who do people go to for help with these problems in the community?*

At the end of the interview:

Would the interviewee be suitable for key informant interview? yes/no

| Problem reported by  interviewee | For psychosocial problems:  a) Who is knowledgeable about these problems in the  community?  b) Who do people go to for help with these problems in the community? |
| --- | --- |
